# A new fatigue damage model for pavement concrete beams bearing multi-level bending loads

**DOI:** 10.1371/journal.pone.0255048

**Published:** 2021-08-05

**Authors:** Liang Lei, Shi Xingang, Cui Yunhua, Wang Lefan, Yan Xiangcheng

**Affiliations:** 1 Chang’an University Shaanxi, Xi’an, China; 2 Air Force Engineering University Shaanxi, Xi’an, China; 3 Engineering Design Department of the Army Research Institute, Beijing, China; China University of Mining and Technology, CHINA

## Abstract

MTS-810 material testing machine and acoustic emission signal analyzer were adopted to explore the mechanical behavior of concrete beams broken by the static load and the nonlinear cumulative damage law of concrete beams broken by fatigue bending from single-stage loading. Then, by introducing the Ramesh Talreja’s Damage Criterion, the damage rule of single-stage loading was extended to the damage accumulation rule under multi-stage loading, and the results were verified by the results of two-stage and three-stage fatigue loading tests. Two main conclusions are achieved: first, affected by four-point bending load, the fatigue life of the concrete specimen is in line with the law of the two-parameter Weibull distribution, namely the higher the stress level is, the shorter the fatigue life is. Second, an obvious nonlinear relationship was discovered in the damage of concrete. The model deduced in this paper and the Palmgren-Miner linear damage accumulation model were adopted to compare the test results of flexural fatigue under single, two and three stage loads. The calculation results of this model were more reliable.

## 1. Introduction

The airport pavement is subjected to multi-level cyclic loads because the load characteristic of the new aircraft on the pavement are different in each stage of take-off and landing. And the current fatigue equations of rigid pavement in China are mainly based on concrete beam flexural fatigue experiments. However, the existing beam flexural fatigue equations often assume that the applied load is a constant amplitude repeated load, which often ignores the multi levels of time history. Therefore, in view of the mechanical characteristics of airport pavements, the fatigue damage mechanism of concrete bearing multi-level loads needs to be further explored.

In terms of the fatigue failure of pavement concrete, previous studies mainly revolved around fatigue theory and experimental results of concrete beams bearing constant amplitude loads indoors [1–4]. The theory of fatigue damage accumulation, with damage accumulation and evolution as its core, originally originated from the research results of metal fatigue damage. Now, studies focus more on the mode of damage accumulation, the implication of damage amount and the definition of critical damage variables. In 1945, based on Palmgren’s study and a large number of experiments [5], Miner proposed the linear cumulative damage theory. It is soon widely applied to practical engineering because of its concise and easy-to-understand form. However, the cumulative damage factor introduced by the Miner criterion is not the same as the actual damage. The cumulative damage factor is an abstract ideal concept while the damage amount is a true reflection of the actual structural mechanical properties. Different damage indicators reflect different situations. Further analysis showed that the Miner criterion simultaneously implied three assumptions: (1) The loading sequence had nothing to do with the law of cumulative fatigue damage. (2) At the same stress level, the amounts of damage generated by each load were the same. (3) The damage level of the current component was unrelated with the loading experiencing. Shah proposed a cubic polynomial cumulative damage model based on characteristics of the three-stage development of fatigue damage [6]. At the same time, Holmen et al. conducted a large number of fruitful experimental studies on the compressive fatigue performance of coagulation. The results showed that variable amplitude loading and random loading accelerated component fatigue damage, while small loads in the loading process slowed down the effect of loading sequence [7,8]. Based on the cubic polynomial cumulative damage model, proposed by B.H.Oh, the nonlinear cumulative damage characteristics of concrete bearing bending action are further verified by theoretical and experimental calculation [9–11].

Recently, scholars have done some research on fatigue concrete models [12,13]. Samuel derived a damage model as a function of expended life fraction of applied load, the logarithm life of applied load and the logarithm life of the initial applied load [14]. Jia evaluated the evolution of reliability with number of cycles for asphalt mixture bearing repeated fatigue loading [15]. Ashish Aeran proposed a new fatigue damage model, based on the commonly available *S*-*N* curve parameters of the material and does not require any additional material parameter determination or *S*-*N* curve modification [16]. Indra studied a phenomenological fatigue damage based on the continuum damage mechanics theory of concrete materials bearing fatigue loading, which can describe the damage evolution of concrete materials bearing different fatigue loading by verifying the predicted fatigue life of concrete materials in uniaxial compression loading [17]. Rafiqul compared traditional stiffness and energy based fatigue failure criteria with the fatigue failure criterion based on the viscoelastic continuum damage approach [18]. By introducing the load weight function and taking residual strength as damage variable, Wang established the cumulative damage curve equation of damage variable and fatigue action times [19]. Song studied the effects of maximum and minimum load level on the evolution strain rate, energy dissipation, acoustic emissions and P-wave speed are analysed. Based on particle based numerical simulations, damage models corresponding to single-level and multi-level cyclic loading tests are proposed [20,21]. Zhu defined the fatigue damage with maximum fatigue strain, residual strain and transverse supersonic wave velocity, respectively. The rationality of Corten-Dolan residual fatigue life model was verified [22,23].

At present, despite the intensive understanding of the cumulative damage of pavement concrete beam, the scholars haven’t reached a consensus on the law of nonlinear cumulative damage. First, the definition of damage lacks a unified evaluation index. The residual stiffness, residual strength, residual strain and cumulative damage factor measured by various test methods were used as the damage values. As a result, the damage curves were not completely consistent. Second, as for the damage accumulation method, the existing test rules and theoretical results are mainly based on the uniaxial tensile test and compression test of concrete, while the damage accumulation rule for the bending fatigue test of concrete beams still needs to be explored. Third, considering the randomness and complexity of concrete composition, the definition of critical damage as a random variable still requires a lot of experimental studies. Fourth, concrete, separately bearing uniaxial compression, uniaxial tension, repeated tension-compression, bending fatigue, will exhibit different fatigue characteristics. Therefore, based on the understanding of the influencing factors of fatigue performance, the paper conducted an experimental research and analysis on the fatigue damage of concrete beams bearing bending, which would contribute to the theoretical and experimental references for the further studies.

## 2. Materials and experiment preparation

### 2.1 Materials

We set the flexural tensile strength of airport pavement cement concrete as the main index and the compressive strength as the reference index. The flexural tensile strength of 28d was not less than 5.0 MPa were required in raw materials preparation and mix ratio determination, and the strength grade of the cement concrete was not less than the compressive strength requirements of C30.

#### (1) Cement

Qinling brand 42.5R Portland cement in Yaoxian County, Shaanxi was selected for the test. The cement performance index parameters are shown in Table 1.

**Table 1:** Cement performance index parameters.

Density/(g/cm^3^)	Water requirement for standard consistency/%	80 μm sieve residue/%	Stability	Setting time/h		Flexural strength/MPa		Compressive strength/MPa	
			Boiling	Initial setting	Final setting	3d	28d	3d	28d
3.21	28.3	1.4	Up to standard	2.17h	5.2h	5.4	8.9	30.8	51.1

#### (2) Coarse aggregate

Jingyang limestone gravel was chosen as concrete coarse aggregate in the test. The aggregate density and bulk density were 2.73 g/cm^3^ and 1.64 g/cm^3^, respectively. The surface was rough with multiple edges and corners and the crushing index was 11.2%. The mud content was 0.1% after washing. Since the concrete beam with the size of 400 mm × 100 mm × 100 mm were chosen for the fatigue test, in order to reduce the discreteness of the data, coarse aggregate with the size of 5 ~ 10 mm and 10 ~ 20 mm were selected, the ratio of which was 40:60.

#### (3) Fine aggregate

With the density of 2.65 g/cm^3^ and bulk density of 1.61 g/cm^3^, fine aggregate is selected from the Bahe River medium sand, Ⅱ district. The fineness modulus was 2.81. After washing and drying, the mud content reached 1.1%.

#### (4) Water

The water used for mixing, aggregate washing and curing of concrete specimens is drinking water in Xi’an. In order to prevent the influence of corrosion on fatigue performance, the technical indexes of test water were strictly controlled. And the PH value, chlorine ion content, sulfate ion all met the requirements of the technical specifications after inspection [24].

#### (5) Mix ratio design

Concrete combinations are shown in Table 2.

**Table 2:** Laboratory mix ratio.

Cement	Water	Medium stone	Small stone	Sand
340	149.6	805	537	634

Put the concrete girder into a standard curing room (curing temperature 20 ± 2°C, humidity ≥ 95%). Cured for 180d.

### 2.2 Test methods and equipment

#### (1) Loading equipment

**Fig 1:**
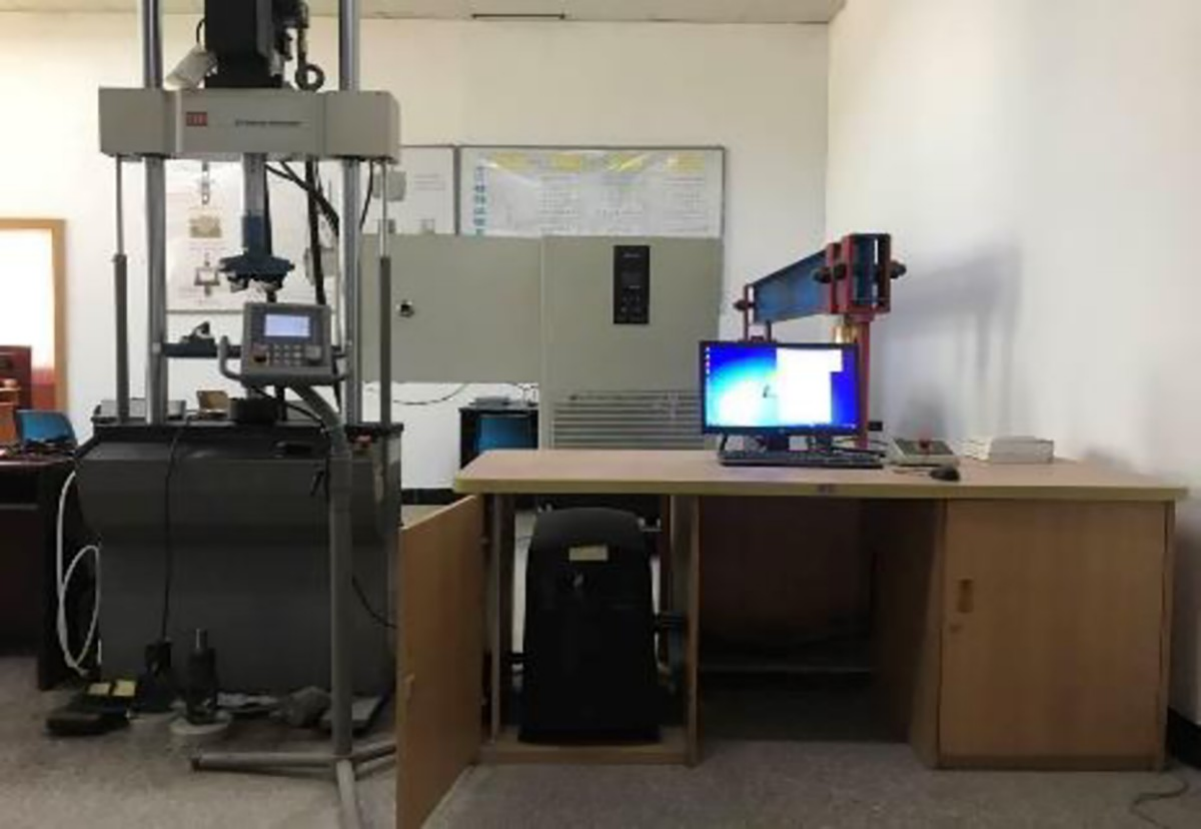
MTS-810 material testing machine.

MTS-810 material testing machine was adopted for loading in the test, as shown in Fig 1. The maximum load of the machine can reach 10 tons, with high-precision displacement, load sensor and two-channel output signal and a hydraulic fixture. Therefore, force control, displacement control, tension test, fatigue test and so forth can be achieved.

Through Station Builder adopted by the MTS-810 material testing machine, the required modality could be respectively established to control the input and output data, and the existing resources of the system could be assembled to obtain the required test data. According to the basic configuration obtained by the Station Builder, the control of the loading unit was strengthened by the Station Manager. Basic test software and multi-purpose test software were adopted to control the test process under normal or irregular loading. Finally, the measured test data was output in excel table for further processing. In the experiment, the basic test software was adopted to control the single-stage fatigue loading and the multi-purpose test software was adopted to control the multi-stage fatigue loading.

#### (2) Acoustic emission damage detection

Eight channels DS5-8A full information acoustic emission signal analyzer, produced by Beijing Soft Island Times Technology Co., were used in the test, which mainly composed of four parts: the signals collection device, sensor, preamplifier and signal analysis software. The sensor was a wideband acoustic emission sensor composed of ceramic piezoelectric element, protective film, shell and damping block. The main technical indicators of signal acquisition instrument are shown in Table 3.

**Table 3:** Technical indexes of DS5-8A full information AE signal analyzer.

Waveform data pass rate/(MB/s)	Continuous data pass rate/(MB/s)	The sampling rate/M	A/D conversion error/LSB	A/D conversion accuracy
48	65.5	5~6	≤±0.5	16 bits

**Fig 2:**
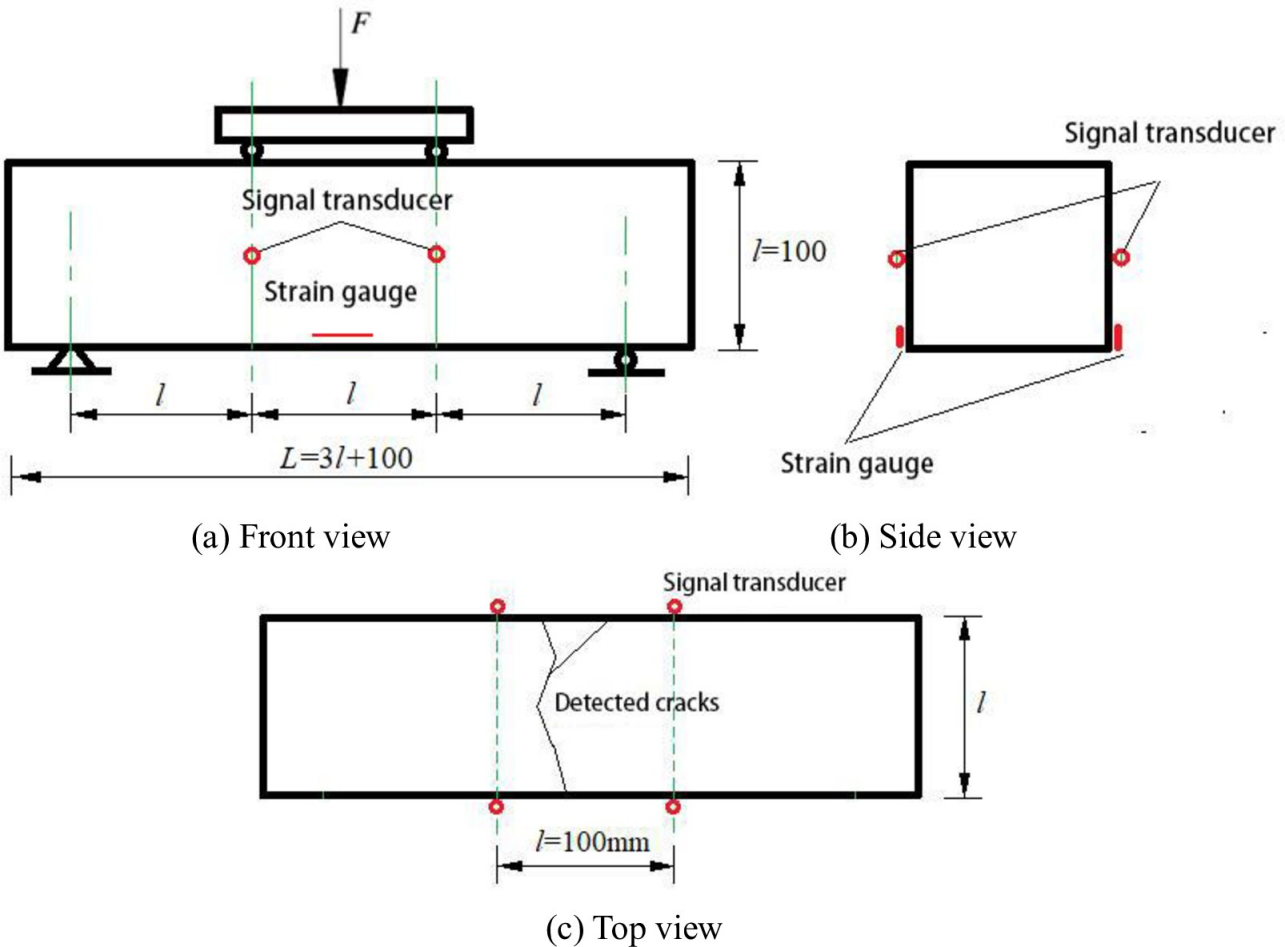
Fixed position of strain gauge and sensor. (a) Front view. (b) Side view. (c) Top view.

The acoustic emission sensors were closely arranged, with a distance of 100 mm from each other, which were pasted on the neutral axis of the concrete beam through a coupling agent (high vacuum silicone grease), as shown in Fig 2.

## 3. Experiment

More than 100 specimens of 400 mm × 100 mm × 100 mm concrete non-standard beams were prepared. The specimens, cured for 6 months under standard conditions, were respectively applied to the fatigue damage test research under single-stage and multi-stage loading. The test adopted the MTS-810 material testing machine to load through four-point bending. The loading waveform was a sine wave and the loading frequency was 5 Hz. The stress ratio R = 0.1.

### 3.1 Static load failure test

The 180d flexural tensile strength of concrete beam was measured by the static load failure test of concrete beam. A 400 mm × 100 mm × 100 mm non-standard concrete beam was adopted to carry out 9 groups of experiments to test the ultimate bending strain and peak stress, mid-span displacement and peak load of concrete beams.

### 3.2 Single-stage loading

Fatigue tests of concrete beams were carried out under constant amplitude loading with four bending points. Three separate stress levels of 0.65, 0.75, and 0.85 were adopted in the experiments of single-stage loading. Six beams experiments were carried out for each stress level, and a total of 18 beams experiments were carried out. The cyclic loads are shown in Table 4.

**Table 4:** Single-stage cyclic load for fatigue test.

Stress level	Peak stress/MPa	Peak load/kN	Valley stress/MPa	Valley load*/*kN
0.65	3.972	13.238	0.397	1.324
0.75	4.583	15.275	0.458	1.528
0.85	5.194	17.312	0.519	1.732

In the process of cyclic loading on fatigue, the damage accumulation law of concrete bearing different stress levels could be analyzed by detecting the residual strength and acoustic emission signal energy after fatigue loading of the four-point bending. The fatigue life during single stage loading was analyzed as the fatigue life calibrated by the specimen bearing multi-stage fatigue loading.

#### (1) Acoustic emission damage detection

**Fig 3:**
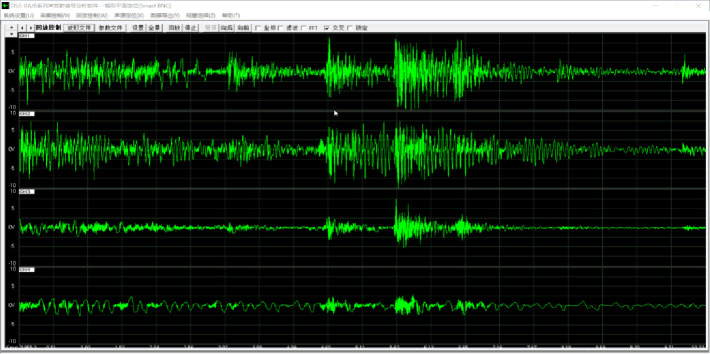
Typical acoustic emission burst signal.

It can be seen from the bending test of static load failure of the concrete that when the specimen is deformed under stress, the deformation can be released in the form of stress wave in a certain proportion and received by the sensor in the form of electrical signal, and the signal strength changes according to the severity of the damage. The acoustic emission signal processed by the terminal is the electrical signal converted from the acoustic signal. The area under the waveform envelope detected by the event signal is defined as energy, as shown in the Fig 3. The cumulative energy in the process of constant amplitude fatigue loading was set as *w*_*as*_, and the total cumulative energy in the process of constant amplitude fatigue loading to failure was set as *w*_*a*_. The relative damage energy *W*_*a*_ = *w*_*as*_/*w*_*a*._

#### (2) Residual strength test

Relative damage strength and relative damage energy are defined as the evaluation indexes of concrete cumulative damage from flexural fatigue. Relative damage strength (*F*_*s*_) refers to the difference value between the average bending strength (*f*_*f*_) and the strength value (*f*_*fs*_), obtained by strength test after constant amplitude loading for a certain number of times, to the *f*_*fs*._ Namely, *F*_s_ = 1-*f*_*fs*_/*f*_*f*._

The fatigue failure times of concrete beams at three stress levels (0.85, 0.75, 0.65) are *N*_f0.85_, *N*_f0.75_, and *N*_f0.65_, respectively. After loading 0.2*N*_fs_, 0.4*N*_fs_, 0.6*N*_fs_, and 0.8*N*_fs_, the residual strength of the specimens was tested, with 3 beam specimens in each group.

### 3.3 Two-stage loading

According to the stress level *S* in low-to-high order (0.65–0.75, 0.65–0.85, 0.75–0.85) and in high-to-low order (0.75–0.65, 0.85–0.65, 0.85–0.75), two level cyclic loading experiments were carried out. In order to reduce the discreteness of test data and screen out of short life test pieces, the first-level load in the circulating load block number is 80% of the corresponding fatigue life, namely *N*_1_ = 80% *N*_*f*1._ The Second load was applied until the specimen cracking. In this process, the cumulative damage factor *D* (*D* = ∑*N*_*i*_/*N*_*fi*_, where *i* was the loading order) was calculated respectively, and the accumulation law of concrete fatigue damage in different loading orders was also analyzed.

### 3.4 Three-stage loading

The three-stage cyclic loading test was carried out in accordance with the sequence of low-to-high stress level S (0.65–0.75–0.85) and high-to-low stress level S (0.85–0.75–0.65). In the cyclic load block, the first-stage *N*_1_ = 20%*N*_*f*1_ was loaded, followed by the second-stage loading, *N*_2_ = 20%*N*_*f*2_, followed by the third-stage loading until the specimen broke. In this process, the cumulative damage factor D (*D* = ∑*N*_*i*_/*N*_*fi*_, where *i* is the loading order) is calculated respectively, and the cumulative law of concrete fatigue damage under different loading orders is also analyzed.

In summary, the fatigue test scheme of concrete trabecular beam is shown in the Table 5.

**Table 5:** Fatigue test scheme of concrete beam.

Loading method	Stress ratio	Group	Number	Total
Static load	——	1	9	9
Single-stage loading	0.85,0.75,0.65	3	6	3×6 = 18 (acoustic emission damage detection)
		4	3	3×4×3 = 36 (residual intensity)
Two-stage loading	0.85–0.65, 0.75–0.65, 0.85–0.75,	3	5	15
	0.65–0.85, 0.65–0.75, 0.75–0.85	3	5	15
Three-Stage loading	0.85–0.75–0.65	1	5	15
	0.65–0.75–0.85	1	5	15

## 4. Results and discussion

### 4.1 Analysis on static loading test

As the 400 mm × 100 mm × 100 mm non-standard concrete beam is adopted, it needs to be converted through the dimension coefficient (0.85), and the result data are listed in the Table 6. Some experimental data are not listed because the fracture location is not within the range of the strain gauge.

**Table 6:** Bending resistance of concrete beams.

Peak stress/MPa	Ultimate bending strain/με	Peak load/kN	Central displacement/mm
6.38	188	25.02	0.076
6.06	159	23.76	0.068
5.78	157	22.67	0.058
6.22	/	24.39	0.063
6.13	162	24.04	0.071
5.99	159	23.50	0.069
6.29	/	24.67	0.074
6.23	168	24.43	0.072
5.87	153	23.02	0.067
Mean value			
6.11	163.72	23.94	0.069
Variance			
0.20	11.66	0.78	0.006
Variable coefficient/%			
3.25	7.12	3.25	8.11

### 4.2 Analysis on fatigue life results from single-stage loading test

The fatigue life of concrete beams bearing three stress levels was obtained through six parallel tests with equal amplitude loading of four-point bending. Due to the large discreteness of the test data, it is necessary to consider the distribution law of fatigue life of specimens while studying the fatigue life under the same cyclic load. At present, common logarithmic normal distribution and Weibull distribution in concrete fatigue life research are commonly used. However, the applicability of boundary conditions of logarithmic normal distribution is insufficient. When logarithmic life is taken to minus infinity, the survival rate of specimens reaches 100%, which is not consistent with the actual situation. Weibull distribution of fatigue life avoids this defect, which can be expressed as:

$$F(N)=P(N_{x}\leq N)=1-\exp[-{\left(\frac{N}{N_{a}}\right)}^{\theta }]$$ (1)

where *N* is the number of cyclic loading. *N*_*x*_ is the random variable corresponding to fatigue life. *N*_*a*_ is the characteristic life parameter. *θ* is the Weibull shape parameter.

Assuming that the bending fatigue life of concrete obtained by the test was a random variable and obeyed the three-parameter Weibull distribution, the probability density function could be expressed as:

$$f(N)=\frac{\theta }{N_{a}-N_{0}}{\left(\frac{N-N_{0}}{N_{a}-N_{0}}\right)}^{\theta -1}\exp[-{\left(\frac{N-N_{0}}{N_{a}-N_{0}}\right)}^{\theta }]\;0<N<\infty$$ (2)

where *N*_*0*,_ *N*_*a*,_ *θ* represent the minimum life parameter, characteristic life parameter and Weibull shape parameter respectively. The failure probability function *F*(*N*_*f*_) and survival probability function *R*(*N*_*f*_) can be expressed as:

$$F(N_{f})=P(N_{x}\leq N_{f})=\int_{N_{0}}^{N_{f}}f(N)dN=1-\exp[-{\left(\frac{N_{f}-N_{0}}{N_{a}-N_{0}}\right)}^{\theta }]$$ (3)

$$R(N_{f})=1-F(N_{f})=\exp[-{\left(\frac{N_{f}-N_{0}}{N_{a}-N_{0}}\right)}^{\theta }]$$ (4)

Take the log of both sides to get the transformation:

$$\ln\{-\ln[1-F(N_{f})]\}=\theta [\ln(N_{f}-N_{0})-\ln(N_{a}-N_{0})]$$ (5)

If ln{-ln[1-*F*(*N*_*f*_)]}, ln(*N*_*f*_-*N*_0_), -*θ*ln(*N*_*a*_-*N*_0_) and *θ* are treated as *Y*, *X*, *a* and *b* respectively, Eq (5) can be converted into a linear equation:

Y=a+bX (6)

The linear regression is used to test the Weibull distribution of fatigue life data. When the default minimum fatigue life of the specimen is 0, that is, *N*_*0*_ = 0, the fatigue life of the specimen is subject to the two-parameter Weibull distribution.

**Fig 4:**
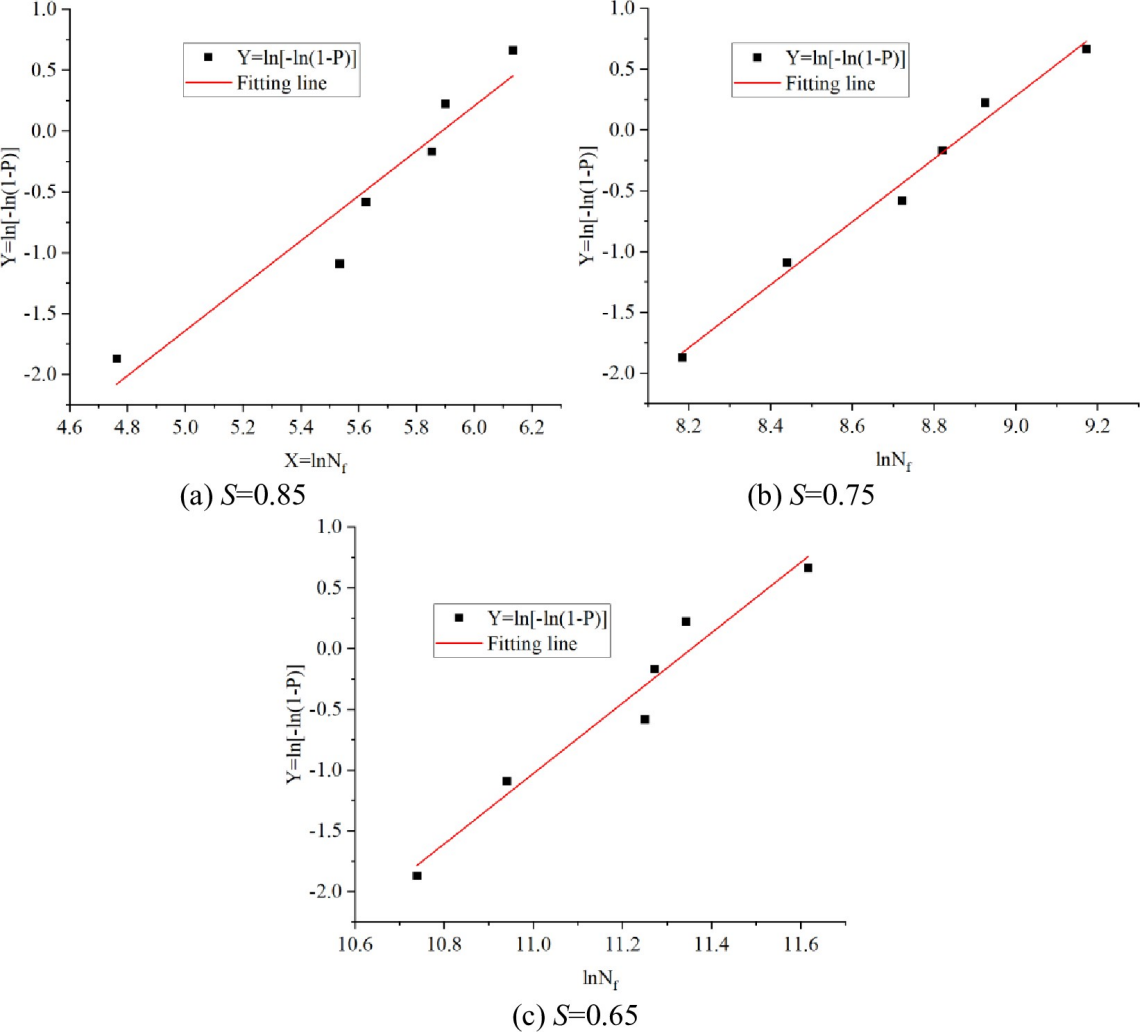
Weibull distribution test for bending fatigue life of specimens. (a) *S =* 0.85. (b) *S* = 0.75. (c) *S* = 0.65.

The failure probability *N*_*f*_ corresponding to fatigue life at different stress levels is calculated by the maximum likelihood method, as shown in Eq (7):

$$P=\frac{i}{K+1}$$ (7)

where *K* is the total number of specimens. *i* is the ordinal number of fatigue life from small to large. When the minimum life parameter is zero (*N*_0_ = 0), the double-parameter Weibull distribution test of the specimen is calculated according to Table 7, as shown in Fig 4.

**Table 7:** Weibull distribution test for bending fatigue life of specimens.

Stress level S	Specimen ordinal	Fatigue life *N*_*f*_/time	Failure probability *P*	*X* = ln*N*_*f*_	*Y* = ln[-ln(1-*P*)}
0.85	1	117	0.1429	4.7622	-1.8698
	2	253	0.2857	5.5334	-1.0892
	3	277	0.4286	5.6240	-0.5805
	4	348	0.5714	5.8522	-0.1657
	5	365	0.7143	5.8999	0.2254
	6	461	0.8571	6.1334	0.6657
0.75	1	3582	0.1429	8.1837	-1.8698
	2	4628	0.2857	8.4399	-1.0892
	3	6132	0.4286	8.7213	-0.5805
	4	6764	0.5714	8.8194	-0.1657
	5	7512	0.7143	8.9243	0.2254
	6	9621	0.8571	9.1717	0.6657
0.65	1	46098	0.1429	10.7385	-1.8698
	2	56395	0.2857	10.9401	-1.0892
	3	76894	0.4286	11.2502	-0.5805
	4	78554	0.5714	11.2715	-0.1657
	5	84301	0.7143	11.3421	0.2254
	6	110863	0.8571	11.6161	0.6657

The regression parameters at various stress levels are listed in Table 8. It can be seen that *X* = ln*N*_*f*_ and *Y* = ln{-ln[1-*P*]} show a good linear relationship and the correlation coefficient *R*^2^ is close to 1, indicating that the fatigue life of the concrete specimen under the action of four-point bending load obeys the two-parameter Weibull distribution.

**Table 8:** Regression parameter results.

Stress level *S*	Regression coefficient *b*	Regression coefficient *a*	Correlation coefficient R^2^
0.85	1.8473	-10.8772	0.9200
0.75	2.5911	-23.0371	0.9879
0.65	2.8984	-32.9107	0.9575

**Fig 5:**
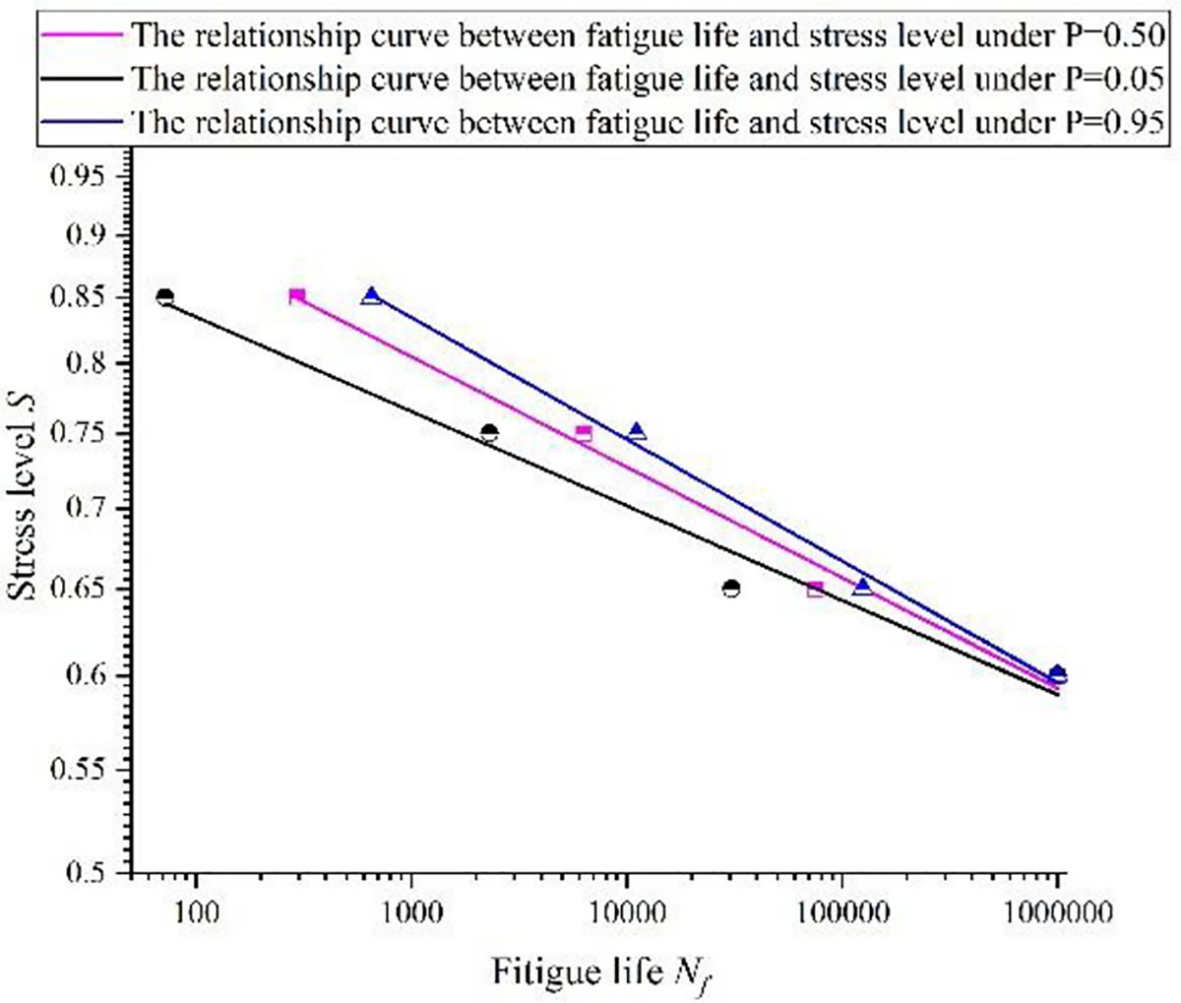
The relationship curve between fatigue life and stress level with different failure probability.

The results of regression parameter show that if the failure probability P = 50%, the bending fatigue life of concrete, bearing the separate stress level of 0.85, 0.75, 0.65, is 296 times, 6307 times and 75,232 times, respectively. After calculating the flexural fatigue life of concrete with failure probability P = 5% and P = 95%, the concrete double-logarithmic fatigue relationship curve is drawn, as shown in Fig 5.

Due to the discreteness of fatigue life data, the results of fatigue life calculated with different failure probabilities differed greatly. It was more dangerous to adopt the calculated life of 0.95 failure probability to represent the fatigue life of specimens, while it was more conservative to adopt the calculated life of 0.05 failure probability to represent the fatigue life of specimens. Therefore, it was appropriate to choose the relationship curve of 0.50 failure probability as the fatigue equation in the test, as shown in Eq (8).


$$\mathrm{lg}\;S=-0.04421\mathrm{lg}\;N_{f}+0.0384$$ (8)

Table 9 shows the test results under different stress ratios. If the number of fatigue action is zero, the residual strength of the specimen will be the concrete flexural strength.

**Table 9:** Residual strength under single stage loading (failure probability P = 0.50, S = 0.85, 0.75, 0.65).

Stress level	Residual strength *f*_*fs*_/MPa				
	0 *N*_*fs*_	0.2 *N*_*fs*_	0.4 *N*_*fs*_	0.6 *N*_*fs*_	0.8 *N*_*fs*_
*S* = 0.85 (*N*_*f*0.85_ = 296)	6.11	5.97	5.58	5.36	4.96
		6.06	5.72	5.44	5.21
		5.86	5.51	5.71	5.17
*S* = 0.75 (*N*_*f*0.75_ = 6307)	6.11	5.96	5.63	5.62	5.14
		6.16	5.79	5.76	5.29
		5.89	5.92	5.39	5.34
*S* = 0.65 (*N*_*f*0.65_ = 75232)	6.11	6.04	5.76	5.69	5.53
		5.98	5.89	5.46	5.48
		6.08	5.94	5.57	5.37

According to the Hypothesis of Ramesh Talreja, the evolution equation of fatigue damage is:

$$1-\frac{N}{N_{fs}}={\left(1-D\right)}^{m+1}$$ (9)

**Fig 6:**
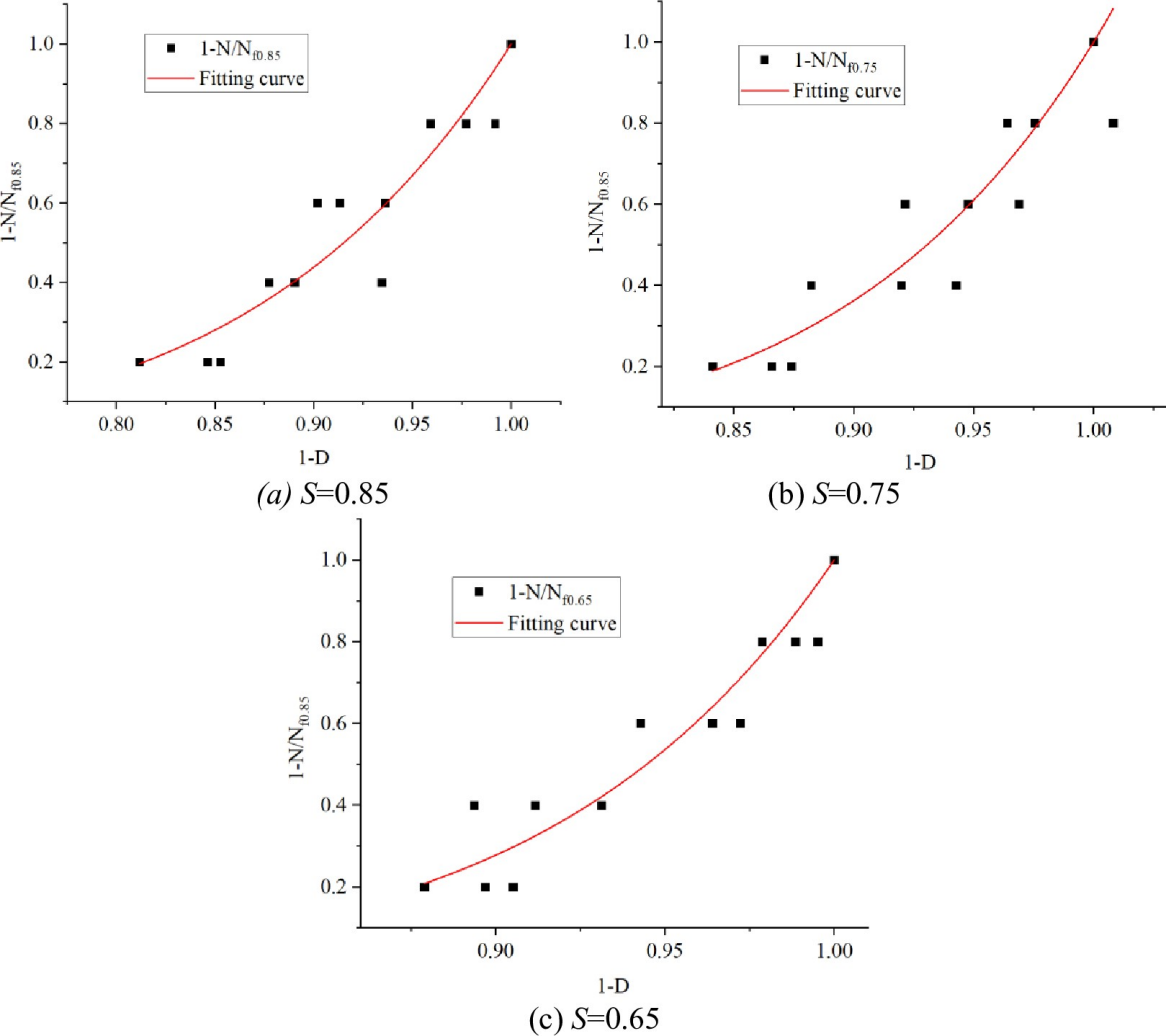
Relative damage strength at different stress levels and test of evolution equation. (a) *S =* 0.85. (b) *S* = 0.75. (c) *S* = 0.65.

The relative damage strength (*F*_s_) was defined as the damage quantity (*D*). The residual strength data measured by the test was converted into the data in Table 10. The evolution curve of concrete damage at different stress levels was plotted, as shown in Fig 6.

**Table 10:** Damage evolution equation test under different stress levels.

*S* = 0.85		*S* = 0.75		*S* = 0.65	
1-*D*	1-*N*/*N*_*f*0.85_	1-*D*	1-*N*/*N*_*f*0.75_	1-*D*	1-*N*/*N*_*f*0.65_
0.8118	0.20	0.8412	0.20	0.9051	0.20
0.8527	0.20	0.8658	0.20	0.8969	0.20
0.8462	0.20	0.8740	0.20	0.8789	0.20
0.8773	0.40	0.9198	0.40	0.9313	0.40
0.8903	0.40	0.9427	0.40	0.8936	0.40
0.9345	0.40	0.8822	0.40	0.9116	0.40
0.9133	0.60	0.9214	0.60	0.9427	0.60
0.9362	0.60	0.9476	0.60	0.9640	0.60
0.9018	0.60	0.9689	0.60	0.9722	0.60
0.9771	0.80	0.9755	0.80	0.9885	0.80
0.9918	0.80	1.0082	0.80	0.9787	0.80
0.9591	0.80	0.9640	0.80	0.9951	0.80
1.0000	1.00	1.0000	1.00	1.0000	1.00

As can be seen from Fig 6, both 1-*D* and 1-*N*/*N*_*fs*_ showed more compliance with the exponential function relationship, and the fitting effect between test data results and damage evolution equation was ideal, that is:

$$D=1-{\left(1-\frac{N}{N_{fs}}\right)}^{1/(1+m)}$$ (10)

After testing, in Eq (10), the regression results of the material parameter m were listed in Table 11.

**Table 11:** Regression parameter results.

Stress level *S*	Regression coefficient *m*	Correlation coefficient R^2^
0.85	6.8256	0.9224
0.75	8.6380	0.8523
0.65	11.1576	0.9067

**Fig 7:**
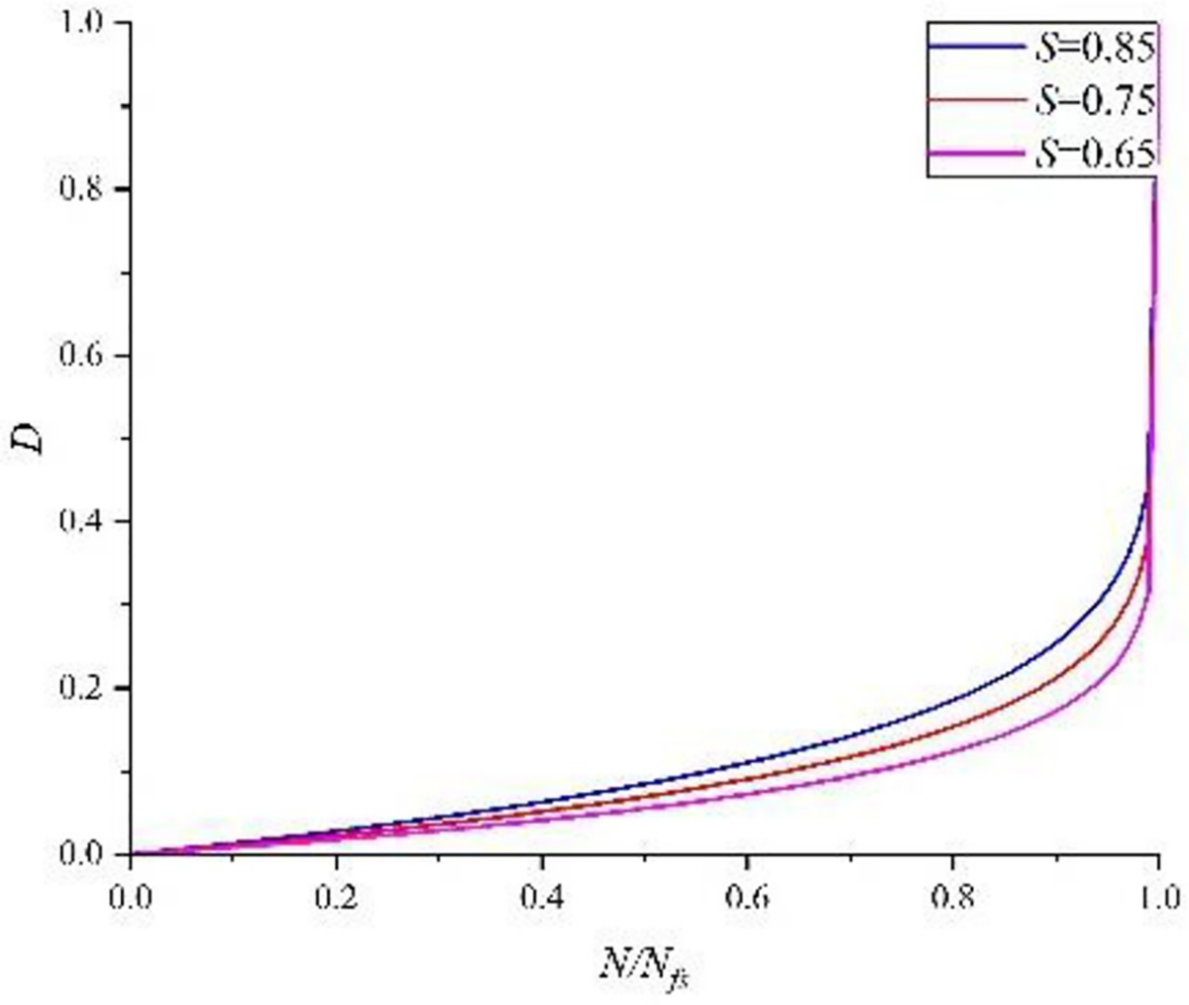
Fitting curve of relative damage strength at different stress levels.

Fig 7 shows the fitting curves at the three stress levels drawn according to the regression coefficient results.

**Fig 8:**
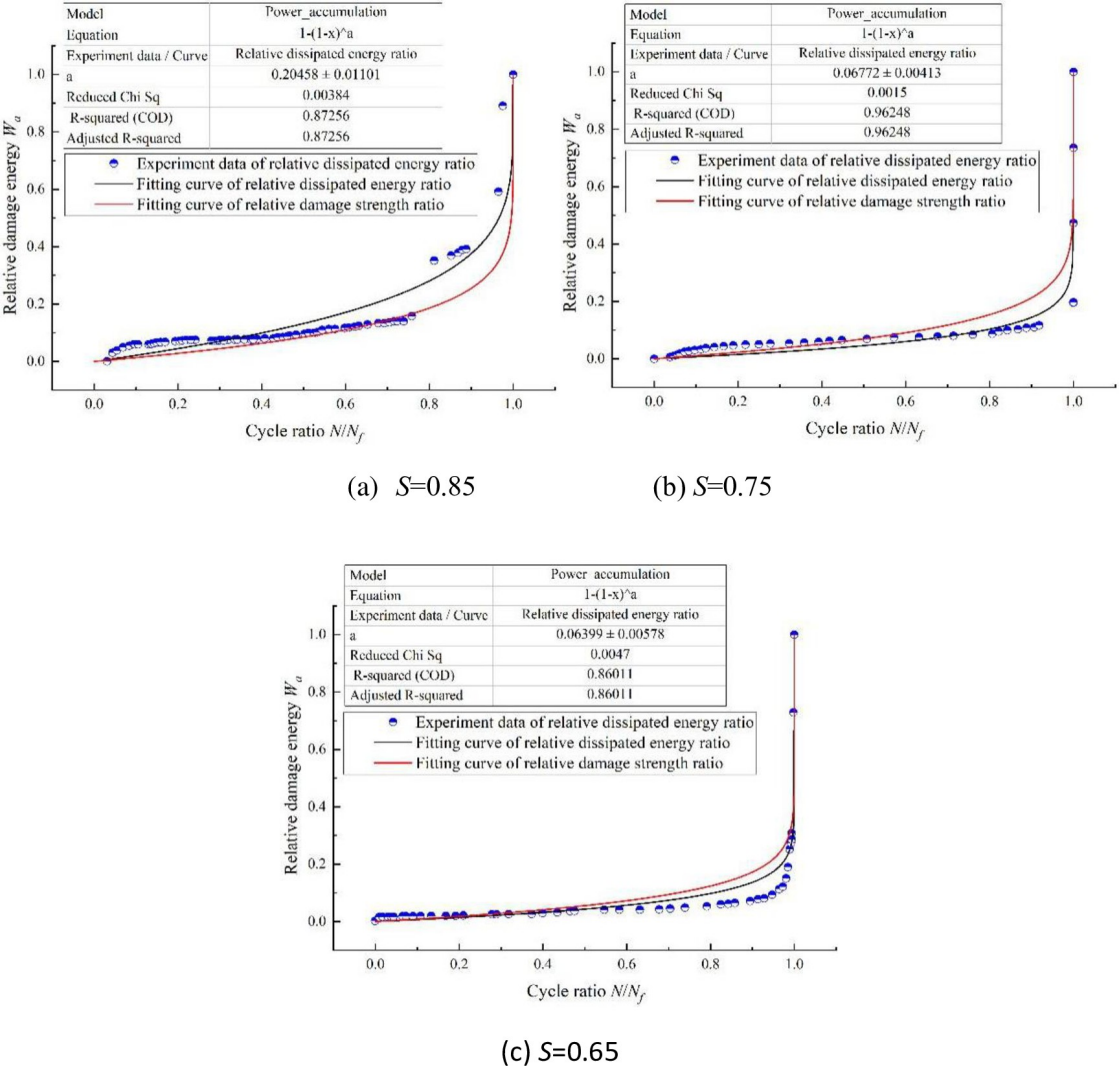
Fitting curve of strength relative damage at different stress levels. (a) *S =* 0.85. (b) *S* = 0.75. (c) *S* = 0.65.

The relative damage energy (*W*_*a*_) refers to the ratio of the accumulated energy (*w*_*as*_) to the total accumulated energy (*w*_*a*_) collected by the acoustic emission signal sensor during the constant amplitude fatigue loading, namely *W*_*a*_ = *w*_*as*_/*w*_*a*_, where the lower Angle *S* = 0.85, 0.75 and 0.65. After fitting the collected acoustic emission signal parameters, the damage law curve as shown in Fig 8 can be achieved.

It can be seen from Fig 8 that under the loading conditions of constant amplitude, constant temperature and fixed frequency, the dissipated energy curves of different stress levels basically conformed to the damage evolution equation. The regression parameter results of the damage evolution equation were shown in Table 12.

**Table 12:** Regression parameter results.

Stress level *S*	Regression coefficient *a*	Correlation coefficient *R*^2^
0.85	0.20458	0.8725
0.75	0.06772	0.9625
0.65	0.06399	0.8601

### 4.3 Analysis of fatigue test results under two-stage load

In order to reduce the discretization of test data and ensure the comparability of test results, the cyclic ratio *N*/*N*_*f*_ = 0.8 was selected as the starting position of grading load. Tables 13 and 14 refer to the flexural fatigue damage test results of concrete at two stages of loading, high to low and low to high respectively. In the table, *i* = 1, 2 and 3 represent the loading sequence of different stress horizontal loads.

**Table 13:** Test results of flexural fatigue damage of concrete under two-stage loading (high-low order).

Stress level *S*	Specimen number	Test data					
		*N*_1_	*N*_1_/*N*_f1_	*N*_2_	*N*_2_/*N*_f2_	∑*N*_*i*_/*N*_*fi*_	Average
0.85–0.65	1	236	0.7973	1369	0.0182	0.8155	0.8883
	2	236	0.7973	3964	0.0527	0.8500	
	3	236	0.7973	5682	0.0755	0.8728	
	4	236	0.7973	10568	0.1405	0.9378	
	5	236	0.7973	12632	0.1679	0.9652	
0.75–0.65	1	5045	0.7999	4564	0.0607	0.8606	0.9542
	2	5045	0.7999	7698	0.1023	0.9022	
	3	5045	0.7999	10984	0.1460	0.9459	
	4	5045	0.7999	15679	0.2084	1.0083	
	5	5045	0.7999	19113	0.2541	1.0540	
0.85–0.75	1	236	0.7973	77	0.0115	0.8088	0.8893
	2	236	0.7973	411	0.0613	0.8586	
	3	236	0.7973	435	0.0649	0.8622	
	4	236	0.7973	729	0.1088	0.9061	
	5	236	0.7973	1432	0.2136	1.0109	

**Table 14:** Test results of flexural fatigue damage of concrete under two-stage loading (low-high order).

Stress level *S*	Specimen number	Test data					
		*N*_1_	*N*_1_/*N*_f1_	*N*_2_	*N*_2_/*N*_f2_	∑*N*_*i*_/*N*_*fi*_	Average
0.65–0.85	1	60184	0.8000	79	0.2669	1.0669	1.3034
	2	60184	0.8000	109	0.3682	1.1682	
	3	60184	0.8000	116	0.3919	1.1919	
	4	60184	0.8000	194	0.6554	1.4554	
	5	60184	0.8000	247	0.8345	1.6344	
0.65–0.75	1	60184	0.8000	564	0.0894	0.8894	1.1264
	2	60184	0.8000	944	0.1497	0.9497	
	3	60184	0.8000	1369	0.2171	1.0170	
	4	60184	0.8000	2953	0.4682	1.2682	
	5	60184	0.8000	4462	0.7075	1.5075	
0.75–0.85	1	5045	0.7999	57	0.1926	0.9925	1.2607
	2	5045	0.7999	98	0.3311	1.1310	
	3	5045	0.7999	113	0.3818	1.1817	
	4	5045	0.7999	188	0.6351	1.4350	
	5	5045	0.7999	226	0.7635	1.5634	

### 4.4 Analysis of fatigue test results under three loads

In the process, because of the early broken of the specimen resulting from the improper setting of cycle ratio, the transformation from a three-stage fatigue loading test into a two-stage loading test should be avoided. Therefore, in the first two stages of loading, the graded cycle ratio was set to 0.2 and the experimental results were shown in Tables 15 and 16.

**Table 15:** Test results of flexural fatigue damage of concrete under three-stage loading (high-low order).

Stress level *S*	Specimen number	Test data							
		*N*_1_	*N*_1_/*N*_f1_	*N*_2_	*N*_2_/*N*_f2_	*N*_3_	*N*_3_/*N*_f3_	∑*N*_*i*_/*N*_*fi*_	Average
0.85–0.75–0.65	1	59	0.1993	1261	0.1999	8126	0.1080	0.5073	0.7402
	2	59	0.1993	1261	0.1999	10667	0.1418	0.5411	
	3	59	0.1993	1261	0.1999	26759	0.3557	0.7550	
	4	59	0.1993	1261	0.1999	34771	0.4622	0.8615	
	5	59	0.1993	1261	0.1999	47933	0.6371	1.0364	

**Table 16:** Test results of flexural fatigue damage of concrete under three-stage loading (low-high order).

Stress level *S*	Specimen number	Test data							
		*N*_1_	*N*_1_/*N*_f1_	*N*_2_	*N*_2_/*N*_f2_	*N*_3_	*N*_3_/*N*_f3_	*∑N*_*i*_/*N*_*fi*_	Average
0.65–0.75–0.85	1	15046	0.2000	1261	0.1999	151	0.5101	0.9101	1.1580
	2	15046	0.2000	1261	0.1999	183	0.6182	1.0182	
	3	15046	0.2000	1261	0.1999	204	0.6892	1.0891	
	4	15046	0.2000	1261	0.1999	276	0.9324	1.3324	
	5	15046	0.2000	1261	0.1999	308	1.0405	1.4405	

**Fig 9:**
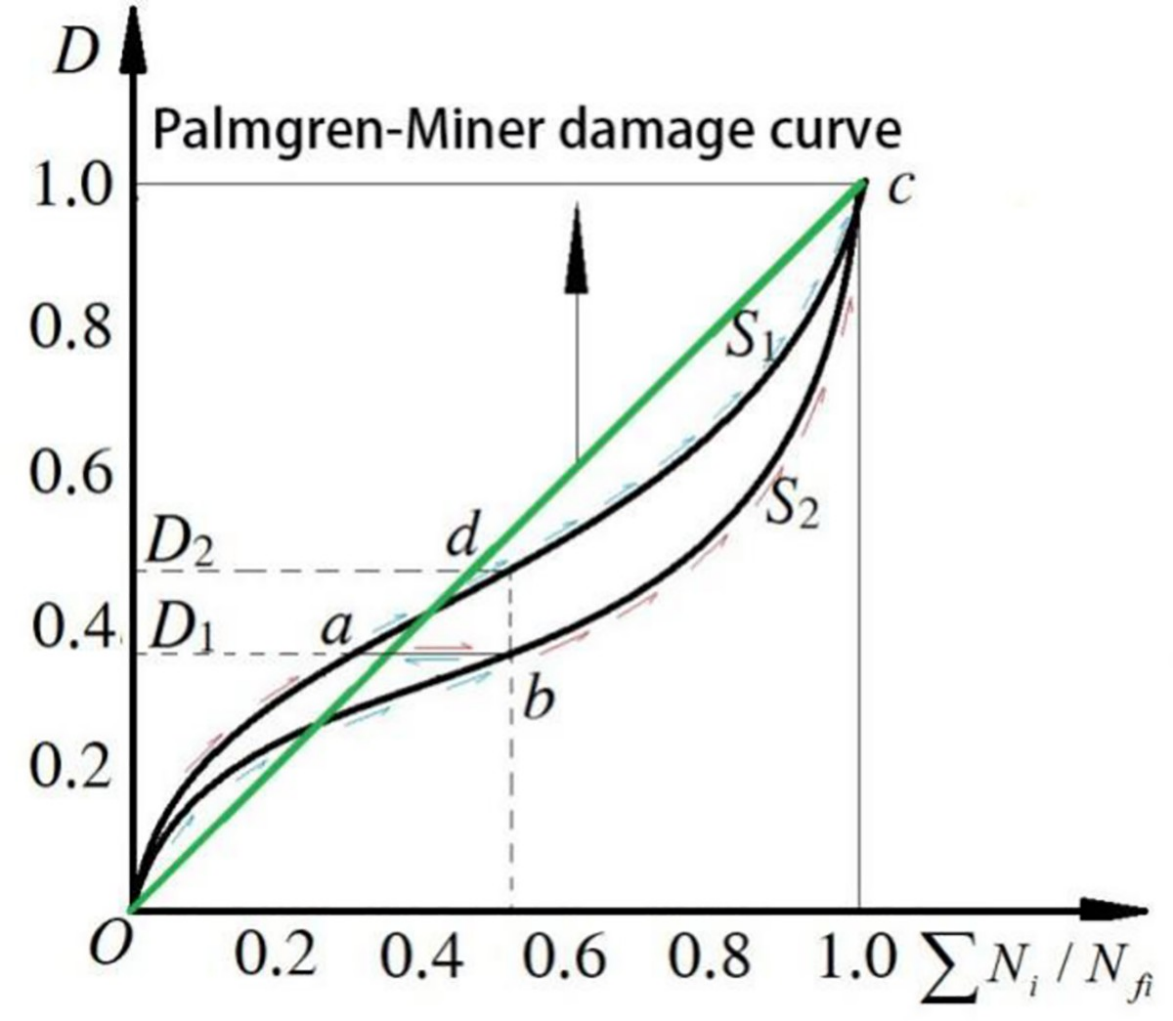
Schematic diagram of fatigue damage development path.

From the test results, it can be seen that the cumulative damage factor (*∑N*_*i*_/*N*_*fi*_) is partially related to the loading order. When the specimen is loaded in high-to-low order, *∑N*_*i*_/*N*_*fi*_*<*1, and vice versa. There are two necessary conditions leading to the cumulative damage result influenced by loading sequence. Firstly, the concrete bearing a single-stage fatigue loading has nonlinear cumulative damage law. Secondly, concrete bearing a different stress level has different damage accumulation curves. With the same cycle ratio, high stress level is accompanied by high damage value, as shown in Fig 9.

As shown in Fig 9, in the process of fatigue loading, the straight line segment *O*→*c* is development-path curve, and *S*_1_ and *S*_2_ curves respectively represent the process of nonlinear fatigue damage development at different stress levels, *S*_1_ and *S*_2_ (*S*_1_>*S*_2_). At the same level of damage amount, the cumulative damage factor D = ∑*N*_i_/*N*_fi_ of fatigue strength *S*_1_ is smaller, which indicates that the Equivalence Hypothesis implied in Miner damage criterion is not correct, that is *D*_1_ = *N*_1_/*N*_f1_ = *N*_2_/*N*_f2_≠*D*_2_. In the process of multistage loading, the development course of fatigue cumulative damage of specimens was also different with different loading sequence. When the specimen suffered from the high-to-low fatigue level, the damage first accumulated along the *O*→*a* curve. At this time, when the fatigue level *S*_2_ was adopted for loading, the damage would remain unchanged along the *a*→*b* curve and then accumulated along the *b*→*c* curve until it was broken, forming the damage accumulation process of *O*→ *a* → *b* → *c*. When the specimen suffered from the low-to-high fatigue level, the damage first accumulates along the *O*→*b* curve. At this time, with the fatigue level *S*_1_ adopted for loading, the damage would remain unchanged along the *b*→*a* curve and then accumulate along the *a*→*c* curve until it is broken, forming the cumulative damage process of *O*→ *b* → *a* → *c*. comparing the development course of *O* → *a* →*b*→ *c* with *O* →*b* → *a* → *c*, we can easily find there is no fatigue effect between the transition from *a* to *b*. Therefore, the number of fatigue actions of the former was reduced and the fatigue life was "shortened". Yet, with the *O* →*b*→ *a*→ *c* damage development course superimposed with the damage accumulation factor of stage *a* to *d*, the fatigue action times increased and the fatigue life became "longer".

Therefore, the Palmgren-Miner linear damage accumulation criterion, which is widely applied in metal materials, is not applicable to brittle materials such as concrete. It is not appropriate to define damage amount by cumulative damage factor.

### 4.5 Prediction and verification of fatigue life

According to the regulation of single-stage fatigue damage, in order to generalize the regulation of two-stage and multistage fatigue damage, the following three assumptions are made for the continuity and equivalence of damage:

1. When the fatigue load is switched between two stress levels, the damage amount remains unchanged, that is, the damage variable is continuous;
2. In the process of multistage fatigue loading, all levels of loading are independent of each other. The rate of damage development has nothing to do with loading history, only with current stress level and current damage amount;
3. When the specimen is damaged, the cumulative value of fatigue damage (equivalent cumulative cycling ratio) is 1.

From the above, it can be seen that if the specimen is damaged successively under *N*_1_ times of load *S*_1_ and *N*_2_ times of load *S*_2_. The fatigue life under two single-stage loads is *N*_f1_ and *N*_f2_ respectively. Then the amount of damage generated by the specimen bearing the first-stage load is:

$$D_{1}=1-{\left(1-\frac{N_{1}}{N_{f1}}\right)}^{k_{1}}$$ (11)

According to the continuity and equivalence of damage, it can be considered that the damage amount *D*_1,_ caused by the cyclic ratio *N*_1_/*N*_f1_ of load *S*_1,_ is equivalent to the damage amount *D*_1_’, caused by the cyclic ratio *N*_2_’/*N*_f2_ of load *S*_2_, then there is:

D1=1−(1−N1Nf1)k1=1−(1−N2'Nf2)k2=D1' (12)

The equivalent cumulative cycle ratio caused by two-stage loads is:

N2'Nf2+N2Nf2=1−(1−N1Nf1)k1/k2+N2Nf2 (13)

It can be seen from hypothesis (3) that, when there are only two stages of load, *N*_2_’+*N*_2_ = *N*_*f*2_. Substituted into Eq (13), then

$$\frac{N_{2}}{N_{f2}}={\left(1-\frac{N_{1}}{N_{f1}}\right)}^{k_{1}/k_{2}}$$ (14)

According to Formula (14), with the criterion of Miner damage, the nominal cumulative cycle ratio caused by two-stage load is:

N1Nf1+N2Nf2=N1Nf1+(1−N2'Nf2)=N1Nf1+(1−N1Nf1)k1/k2 (15)

When *k*_1_/*k*_2_>1, *N*_1_/*N*_*f*1_+*N*_2_/*N*_*f*2_<1. When *k*_1_/*k*_2_<1, *N*_1_/*N*_*f*1_+*N*_2_/*N*_*f*2_>1. When *k*_1_/*k*_2_ = 1, the stress level of the two loads is the same, which can be understood as the fatigue loading at a single stage of load, *N*_1_/*N*_*f*1_+*N*_2_/*N*_*f*2_ = 1. Therefore, Eq (15) better explains the change of different cyclic ratios in different loading orders under multi-stage loads.

By analogy, under three-stage load, the damage amount *D*_2_ from load *S*_1_ and load *S*_2_ is equivalent to the damage amount *D*_2_’ from *N*_3_’ times *S*_3_ load, then there is:

D2=1−[1−(N2'Nf2+N2Nf2)]k2=1−(1−N3'Nf3)k3=D2'N2'Nf2+N2Nf2<1 (16)

Substitute Eq (12) into Eq (16):

N3'Nf3=1−[1−(N2'Nf2+N2Nf2)]k2/k3=1−[1−(1−(1−N1Nf1)k1/k2−N2Nf2)]k2/k3N2'Nf2+N2Nf2<1 (17)

The equivalent cumulative cycle ratio caused by the three-stage load is:

N3'Nf3+N3Nf3=1−{1−[1−(1−N1Nf1)k1/k2−N2Nf2]}k2/k3+N3Nf3 (18)

According to hypothesis (3), when there is only three-stage load, *N*_3_’+*N*_3_ = *N*_*f*3_, then there is:

$$\frac{N_{3}}{N_{f3}}={\left\{1-[1-{\left(1-\frac{N_{1}}{N_{f1}}\right)}^{k_{1}/k_{2}}-\frac{N_{2}}{N_{f2}}]\right\}}^{k_{2}/k_{3}}$$ (19)

In the same way, the equivalent cumulative cycle ratio is

Ni'Nfi+NiNfi=1−[1−(Ni−1'Nfi−1+Ni−1Nfi−1)]ki−1/ki+NiNfi(i=1,2,3,⋯,n)Ni−1'Nfi−1+Ni−1Nfi−1<1 (20)

When the left Eq (20) of equivalent cycle ratio is equal to 1, that is, *N*_*i*_’+*N*_*i*_ = *N*_*fi*_, the fatigue failure of the specimen occurs. Thus the fatigue life can be calculated.

Then, we took the results of fatigue tests loaded with 3 levels in low-to high order as an example to illustrate the prediction model of fatigue life. The stress level of the first-stage load was *S* = 0.65, and the load action *N*_1_ = 15,049. The stress level of the second-stage load was *S* = 0.75, and the load action *N*_2_ = 1,261 times. The stress level of the third load was *S* = 0.85, and the number of remaining life was solved. In the calculation, material parameters *a*_1_, *a*_2_ and *a*_3_ were respectively obtained from the regression parameter results of Table 12.

According to the test results of failure probability *P* = 50%, *N*_f1_, *N*_f2_, *N*_f3,_ affected by the fatigue life of concrete bearing the three different stress levels, can be worked out and they were 296 times, 6,307 times and 75,232 times respectively.

The first stage cumulative cycle ratio N_1_/N_f1_ is equal to 0.2000 and the damage amount from the first-stage load is equivalent to that from the second-stage load:

N2'Nf2=1−(1−N1Nf1)k1/k2=1−(1−0.2000)0.06399/0.06772=0.1901 (21)

The cumulative cycle ratio from two-stage load was calculated as *N*_2_’/*N*_f2_+*N*_2_/*N*_f2_ = 0.1901+0.1999 = 0.39<1. The two-stage equivalent cumulative cycle ratio is less than 1, indicating that the structure is not damaged and can be loaded at the third stage. The cumulative cycle ratio of the damage amount from the action of the first two stages is equivalent to the third stage:

N3'Nf3=1−(1−(N1Nf1+N2'Nf2))k2/k3=1−(1−0.39)0.06772/0.20458=0.8490 (22)

The third-stage cumulative cycle ratio *N*_3_/*N*_f3_ = 1-*N*_3_’/*N*_f3_ = 0.1509 and the remaining life is 45 times.

If Miner damage criterion is used to calculate, there is a third-level cumulative cycle ratio *N*_3_/*N*_*f*3_ = 1-*N*_1_/*N*_*f*1_-*N*_2_/*N*_*f*2_ = 1–0.2000–0.1999 = 0.6001 and the residual life is 178 times.

In order to verify the development law of fatigue damage from single-stage loading and the influence of fatigue loading sequence from multi-stage loading, the following results are obtained in this paper, as shown in Table 17.

**Table 17:** Calculation and verification of fatigue cumulative damage model.

Stress level *S*	Test results ∑*N*_*i*_/*N*_*fi*_	The model in this paper		Palmgren-Miner model	
		*∑N*_*i*_/*N*_*fi*_	Relative dispersion %	*∑N*_*i*_/*N*_*fi*_	Relative dispersion %
Two levels of high—low loading sequence					
0.85–0.65	0.8883	0.8034	9.56	1	-12.58
0.75–0.65	0.9542	0.9821	-2.92	1	-4.8
0.85–0.75	0.8893	0.8054	9.44	1	-12.45
Two levels of low—high loading sequence					
0.65–0.85	1.3439	1.4045	-4.51	1	25.59
0.65–0.75	1.1264	1.0185	9.57	1	11.22
0.75–0.85	1.308	1.387	-6.04	1	23.55
Three levels of high—low loading sequence					
0.85–0.75–0.65	0.7402	0.6892	6.90	1	-35.09
Three levels of low—high loading sequence					
0.65–0.75–0.85	1.158	1.249	-7.86	1	13.65

It can be seen from Table 17 that the cumulative damage factor of Palmgren-Miner model (∑*N*_i_/*N*_fi_) is significantly different from the test value. Under the loading condition of 0.85–0.75–0.65, the maximum relative deviation can reach 35.09%. However, the relative deviation between the calculated results and the test results (the relative deviation refers to the ratio on the calculated value to the test value is relatively small and remains within 10%, which indicates that the nonlinear damage accumulation model is more in line with the concrete fatigue damage behavior of the concrete.

## 5. Conclusions

The paper adopted MTS-810 material testing machine to study the accumulation law of fatigue damage of concrete beam bearing multi-stage loading. The main conclusions are as follows:

1. Affected by four-point bending load, the fatigue life of concrete specimens obeyed two-parameter Weibull distribution. The higher the stress level was, the shorter the fatigue life would be.
2. According to the observation of damage accumulation curve obtained by test results, we can easily find when the fatigue loading cycle was smaller than *N*/*N*_f_<0.8, the damage evolution process was slower. When *N*/*N*_f_ = 0.8, at the stress levels of *S* = 0.85, *S* = 0.75 and *S* = 0.65, the relative dissipated energy only increased by 28.05%, 10.33% and 9.79%, respectively. When the fatigue loading cycle ratio *N*/*N*_f_ is equal to 0.95, the concrete damage was significantly aggravated. The relative dissipation energy reached 45.82%, 18.36%, and 17.44% respectively at the three stress levels of *S* = 0.85, *S* = 0.75, and *S* = 0.65. At the end of loading, the dissipation energy of specimens increased sharply, showing a phenomenon of brittle fracture at macro level.
3. In the process of multistage fatigue loading, cumulative damage factor of concrete is not exactly equal to 1. It has a certain relationship with loading sequence. In high-to-low loading sequence, concrete cumulative damage factor *D* = ∑ *N*_i_/*N*_fi_<1; in low-to-high loading sequence, concrete cumulative damage factor D = ∑*N*_i_/*N*_fi_>1, which further proves the nonlinear relationship of concrete damage.
4. Through formula derivation, the paper extended a single-stage loading into the damage accumulation relation at any stage to verify the final result, and utilized the results of two-stage and three-stage fatigue loading tests. Analysis showed that relative deviations of calculation results, derived from two-stage loading in high-to-low sequence, were 9.56%, 2.92% and 9.44% respectively; relative deviations of calculation results, derived from two-stage loading in low-to-high sequence, were 4.91%, 9.57% and 6.04% respectively; derived from three-stage loading in high-to-low sequence, the relative deviation was 6.90%; derived from three-stage loading in low-to-high sequence, relative deviation was 7.86%. Compared with the Palmgren-Miner linear damage accumulation model, the calculated results of nonlinear model in the paper are more reliable.

## Supporting information

S1 Data (ZIP)
